# Study on Adsorption Properties of Calcined Mg–Al Hydrotalcite for Sulfate Ion and Chloride Ion in Cement Paste

**DOI:** 10.3390/ma14040994

**Published:** 2021-02-20

**Authors:** Jun Wang, Bei Huang, Zhongyang Mao, Yu Wang

**Affiliations:** 1 College of Materials Science and Engineering, Nanjing Tech University, Nanjing 211800, China; 201861203136@njtech.edu.cn (J.W.); mzy@njtech.edu.cn (Z.M.); 201861203018@njtech.edu.cn (Y.W.); 2 State Key Laboratory of Materials-Oriented Chemical Engineering, Nanjing 211800, China

**Keywords:** sulfate attack, chloride attack, magnesia–alumina hydrotalcite, adsorbent

## Abstract

In the marine environment, sulfate ions and chloride ions are abundant. Therefore, sulfate attack and chloride ion attack are common failure forms of marine concrete. Mg–Al hydrotalcite is a layered bimetallic hydroxide, which can be used as guest molecular adsorbent. In this experiment, we synthesized Mg–Al hydrotalcite, and the crystal state, surface morphology, and composition of this adsorbent were investigated by modern micro-analysis technology. Mg–Al hydrotalcite was added into the prepared target ion solution, to explore the influence of various factors on the adsorption performance of Mg–Al hydrotalcite, and then calcined Mg–Al hydrotalcite was added into cement paste, to study the mechanical properties and durability of the paste samples. The experimental results show that the optimum conditions for adsorption of chloride ions by calcined Mg–Al hydrotalcite are an adsorption time of 4 h, temperature of 35 °C, LDO (calcined Mg-Al hydrotalcite) dosage of 3.5 g/L, and a pH of 8. The adsorption effect of sulfate ion is best when the adsorption time is 6 h, the temperature is 35 °C, the dosage of LDO is 4 g/L, and the pH = 8. The optimal adsorption conditions of calcined Mg–Al hydrotalcite for chloride ion and sulfate ion are not completely the same, and the adsorption of these two ions in mixed solution shows competitive adsorption. Compared with the common paste specimens without Mg–Al hydrotalcite, the mechanical properties and deformation properties of cement specimens can be significantly improved by adding Mg–Al hydrotalcite.

## 1. Introduction

Cement concrete is the most widely used building material, but in recent years, many concrete projects have experienced premature failure and collapse. Especially in the marine environment, concrete structures are easily damaged, and the main causes of damage are steel corrosion and salt erosion. Moreover, chloride and sulfate erosion are the biggest culprit. Chloride ion erosion firstly rusts the reinforcement of reinforced concrete, leading to cracks along the reinforcement of concrete; secondly, chloride solution migrates to the concrete surface with the continuous drying of concrete, which generates frost or crystallizes in the pores of the concrete surface and produces crystallization expansion stress, which leads to concrete-surface peeling and cracks [1]. The sulfate attack process of concrete includes many complex chemical reactions, and the products are also very complex, which is accompanied by the physical crystallization expansion of sulfate. On the one hand, the essence of its erosion is that sulfate ions in the environment invade into concrete and react with hydration products to produce ettringite, gypsum, and other expansive substances, and at the same time, it causes cracking and spalling of concrete under the action of sulfate crystallization expansion. On the other hand, the reaction between sulfate and cement hydration products reduces the strength and bonding performance of concrete [2]. At present, scholars from all over the world have done many experiments to explore the durability of concrete. However, most of the researches mainly focus on adding mineral admixtures, to improve the durability of concrete. For the chemical corrosion caused by the infiltration of potentially harmful ions such as chloride ions and sulfate ions, most of the admixtures only slow down the transport of internal ions from the aspect of dense pore structure. There are few studies on improving the durability of concrete from Immobilizing free anions. Hydrotalcite is a layered bimetallic hydroxide. The main structural characteristics are that the laminates of two metal hydroxides are orderly arranged along the axial direction to form three-dimensional crystals, the two metal elements are usually divalent and trivalent, and the atoms are covalently bonded. Anions exist between layers, which are connected with the main layer, by weak chemical bonds, such as ionic bonds and hydrogen bonds. The skeleton of the laminate is positively charged, and the interlayer anions are balanced with it, which is electrically neutral as a whole. Its structural general formula is [M^2+^_1−x_M^3+^_x_(OH)_2_](A^n−^)_x/n_∙mH_2_O. M^2+^ represents a divalent metal cation, M^3+^ represents a trivalent metal cation, x represents the ratio of trivalent metal cations to divalent metal cations, A^n−^ indicates inorganic anions or organic groups between layers, and m is the number of interlayer water molecules [3]. The interlayer anions of Mg–Al hydrotalcite are exchangeable because the interlayer anions of Mg–Al hydrotalcite are connected by hydrogen bonds [4]. Based on this exchangeability, Mg–Al hydrotalcite materials are also widely used as ion exchange materials and adsorption materials [5,6]. Jone [7] used hydrotalcite materials to remove complex anions of some metal ions in solution by using the ion exchange method. As an adsorption material, hydrotalcite materials are now more used in wastewater treatment and other industries. Orthman [8] used it to remove organic dyes from dye wastewater. Jone [7] used calcined hydrotalcite materials to remove Ni(CN)_4_^2−^, CrO_4_^2−^, and other complex anions from some metal ions in solution. Ulibarri [9] used calcined hydrotalcite to remove trichlorophenol and trinitrophenol from wastewater.

In concrete engineering, Tatematsu [10] uses the exchange characteristics of Calcite-boehmite to remove chloride ions and sulfate ions in concrete. Shui Zhonghe [11,12] of Wuhan University of Technology has studied the chloride ion curing ability of magnesium–aluminum nitrate layered double hydroxides (LDHs) and calcined Mg–Al hydrotalcite (LDO) in cement paste. The results show that LDHs can show good chloride-ion-curing ability in a cement environment.

Some previous works in the literature [13,14,15,16,17] focused more on the adsorption of a single ion by LDHs, but they did not study the coupling adsorption of multiple ions. In this experiment, Mg–Al hydrotalcite was synthesized at first. It was theoretically proved by various microscopic means that Mg–Al hydrotalcite has good anion adsorption capacity. The coupling adsorption capacity of Mg–Al hydrotalcite was actually detected in the aqueous solution with multiple ions, and the best adsorption conditions for each ion were explored. Finally, hydrotalcite material was added into cement paste samples, to study the target ion curing ability of hydrotalcite material in the environment of cement hydration products.

## 2. Materials and Methods

### 2.1. Materials

#### 2.1.1. Raw Materials of Preparing Mg–Al Hydrotalcite

In this experiment, magnesium nitrate, aluminum nitrate, urea, anhydrous ethanol and deionized water were used as raw materials to synthesize magnesium–aluminum hydrotalcite (LDHs) by hydrothermal method, and then calcined to produce calcined magnesium–aluminum hydrotalcite (LDO). The purity of all reagents is over 99%.

#### 2.1.2. Prepare Raw Materials of Target Ion Aqueous Solution

In this experiment, firstly, the adsorption capacity of LDO in aqueous solution was studied. Using sodium chloride and sodium sulfate as raw materials, we observed that the purity of both is over 99%. According to different research purposes, different concentrations of separate solutions and mixed solutions were prepared.

#### 2.1.3. Raw Materials of Cement Paste Test Pieces

P·II 52.5 Portland cement was used in this study, and its chemical composition is shown in Table 1. With cement as raw materials, with a water-cement ratio of 0.25, rectangular cement paste specimens of 20 mm × 20 mm × 80 mm were formed. LDO with a cement mass of 3% was added in the experimental group, while LDO was not added in the control group. Two groups of specimens were placed in the mixed solution of sodium chloride and sodium sulfate for curing test specimens of different ages.

### 2.2. Methods

#### 2.2.1. XRD and TG–DSC Analysis

X-ray diffraction analysis of LDHs and LDO was carried out. The test powders were passed through an 0.08 mm sieve. The XRD data were collected in the range of 5°–80°, 2θ at a counting time of 15 s/step and a divergence slit of 1°. TG–DSC data were obtained from 50 to 900 °C, at a rate of temperature increase of 10 °C/min, in an N_2_ atmosphere, in cement experiment.

#### 2.2.2. SEM Analysis

In order to study the micro-morphology of LDHs and LDO, A scanning electron microscope (JSM-6510, Jeol, Tokyo, Japan) was used to observe the surface morphology of LDHs and LDO.

#### 2.2.3. FTIR Analysis

To study the molecular structure and chemical bonds of LDHs and LDO, the Fourier-transformed infrared (FTIR) spectra of each solid product were recorded on a Nexus 670 Spectrometer (Nicolet, Madison, WI, USA). Specifically, a 1 mg sample and 200 mg KBr in a dry environment were pressed in a hydraulic press, to form a tablet, and the FTIR spectrum of the sample was measured with a resolution of 2 cm^−1^, in the wavenumber range of 4000–400 cm^−1^.

#### 2.2.4. AQ Analysis

AQ analysis is a method to detect the ion concentration in solution by ion chromatograph. A certain amount of LDO was added into sodium chloride solution and sodium sulfate solution, and after a period of oscillating adsorption, it was filtered, and the content of chloride ion and sulfate ion in the solution was detected by ion chromatography equipment (Thermo Scientific, Waltham, MA, USA).

#### 2.2.5. Deformation Performance Test of Cement Paste Specimen

**Figure 1:**
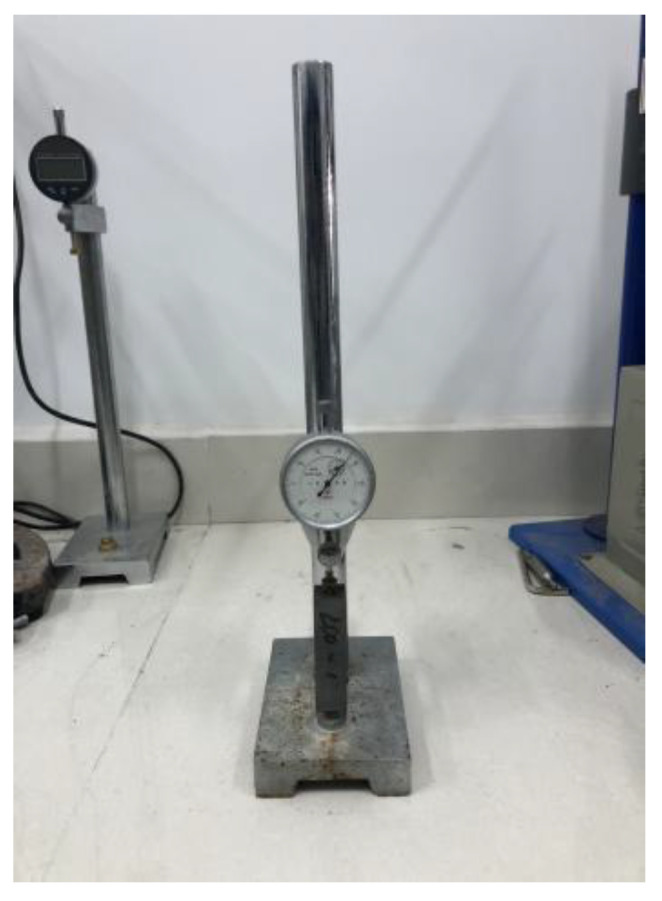
Expansion test on cement paste.

The deformation performance of cement paste specimens at each age was tested. According to Equation (1), the expansion of a specimen at a certain age was calculated by the average of the measurements performed at both sides, with a length comparator with 0.01% precision, as shown in Figure 1. In the formula, *L*_0_, *L*, and *L_t_* represent the initial length, effective length of 80 mm, and testing length at different ages, respectively.

(1) $$P=\;\frac{L_{t}-L_{0}\;}{L}\;\times 100\%$$

#### 2.2.6. Compressive Strength of Cement Paste

**Figure 2:**
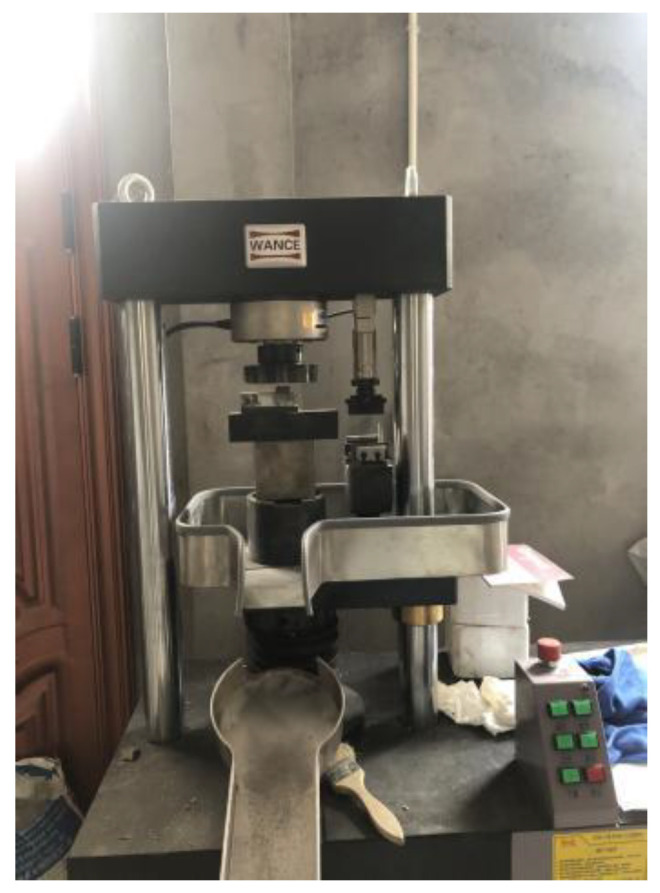
Compressive strength test on cement paste.

The compressive strength of cement paste was examined at different ages, by using a compressive-testing machine with a loading rate of 0.5–0.8 MPa/s. The machine for testing compressive strength is shown in Figure 2. For each test, the average compressive strength of the three paste samples was used. 

## 3. Preparation Method of Magnesium Aluminum Hydrotalcite

According to the ratio of n [CO(NH_2_)_2_]:n (Mg^2+^ + Al^3+^) = 10:1 and n(Mg^2+^):n (Al^3+^) = 2.5:1, 6.4 g Mg (NO_3_)_2_·6H_2_O and 3.75 g Al(NO_3_)_3_·9H_2_O were weighed, and then they were dissolved in 100 mL deionized water. Then, 21 g of CO(NH_2_)_2_ was dissolved in 100 mL of mixed solution of ethanol and deionized water (the volume ratio of ethanol to deionized water was 1:1), and the solution was continuously stirred at 50 °C, for 20 min. The next steps were transferring the mixed solution to a crystallization kettle lined with polytetrafluoroethylene, placing in an oven, crystallizing at a constant temperature of 150 °C for 12 h, taking out the autoclave, and cooling to room temperature. The obtained precipitate was filtered, washed several times with deionized water and absolute ethanol, and then dried at 100 °C, for 6 h, to obtain magnesium–aluminum hydrotalcite (LDHs). Calcined Mg–Al hydrotalcite (LDO) was obtained by roasting LDHs at 450 °C for 4 h. LDO has a “memory effect” of structure. When LDO is added into the aqueous solution containing the required anions, it can absorb anions to restore the original layered structure, thus achieving the goal of removing the target anions. In the process of reconstructing its layered structure, LDO needs more anions, and its adsorption capacity is much larger than that before calcination. Later experiments were carried out with LDO as adsorbent material.

## 4. Results

### 4.1. Characterization of LDHs and LDO

#### 4.1.1. XRD Analysis of LDHs and LDO

**Figure 3:**
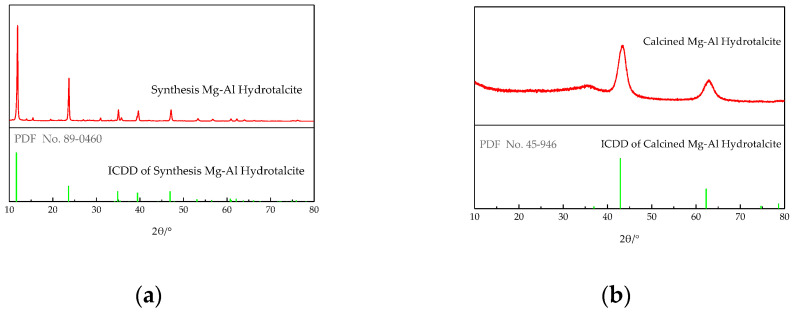
XRD patterns of LDHs and LDO: (**a**) LDHs and (**b**) LDO.

Figure 3a shows the XRD pattern of LDHs (PDF-#89-0460). It can be seen from the figure that the sample has five characteristic diffraction peaks (003, 006, 012, 015, and 018) with high relative diffraction intensity, which appear around 2θ of 11.6, 23.4, 34.8, 39.4, and 47.8, respectively. The full width at half maximum values (FWHM) are 0.237, 0.240, 0.222, 0.338, and 0.308, respectively. The diffraction peak of the product corresponds to Mg_0.667_Al_0.333_ (OH)_2_ (CO_3_)_0.167_ (H_2_O)_0.5_, and the product belongs to the typical layered structure of root cutting carbonate layered hydrotalcite. The width of the peak is narrow and sharp, and there is no obvious impurity peak, which indicates that the product has high purity, complete structure, and single crystal phase. Figure 3b shows the XRD pattern of LDHs calcined at 450 °C (PDF-#45-0946). It can be seen from the figure that the sample has two characteristic diffraction peaks with high relative diffraction intensity, which appear around 2θ of 42.9, and 62.2, respectively. The characteristic peaks belonging to LDHs are obviously weakened or even disappeared, which may be caused by the loss of most interlayer water and anions in the calcined products. The full width at half maximum values (FWHM) are 0.846 and 0.755, respectively. Only the characteristic diffraction peak of MgO oxide appears in the spectrum of this sample, which indicates that, after calcination, the interlayer hydroxyl, carbonate, and H_2_O of LDHs sample are completely lost and become MgO oxide.

#### 4.1.2. The SEM Analysis of LDHs and LDO

**Figure 4:**
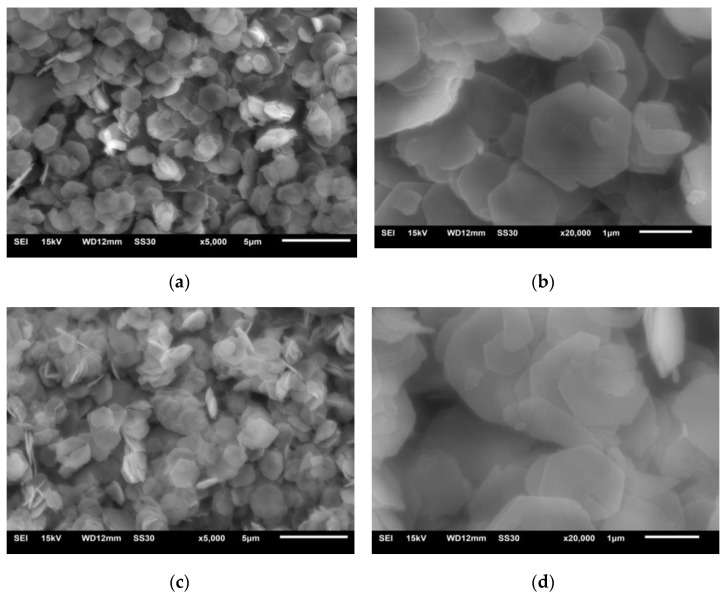
Typical SEM (5000 times and 20,000 times) morphology images of LDHs and LDO. (**a**) LDHs at 5000 times (**b**) LDHs at 20,000 times (**c**) LDO at 5000 times, and (**d**) LDO at 20,000 times.

The special properties of LDHs depend on its microstructure to a great extent, and the morphology of fine structure surface can be clearly observed by scanning electron microscope. Figure 4a,b shows electron micrographs with the magnification of LDHs of 5000 times and 20,000 times, respectively. It can be seen that LDHs synthesized from ethanol are a typical flake hexagonal structure. LDHs synthesized by this method have a smooth surface and a complete crystal with the size of 1~2.5 μm, and some Mg–Al water slides cross-grow to form a supporting structure. Because ethanol has a good dispersion effect, this structure can prevent agglomeration among the slides and improve their dispersibility.

Figure 4c,d shows electron micrographs of LDO with magnification of 5000 times and 20,000 times, respectively. Comparing Figure 4a,b, it can be seen that the morphology of the sample has not changed much after calcination, and it still maintains its original structure. Because of the large cross-sectional area of flaky LDHs, the surface of the sample does not change obviously during the process.

#### 4.1.3. The EDS Analysis of LDHs and LDO

**Figure 5:**
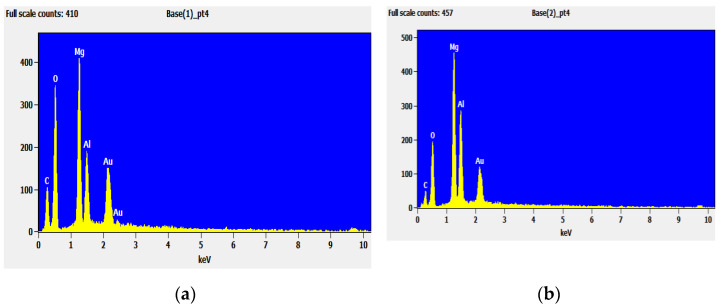
The EDS analysis of LDHs and LDO: (**a**) LDHs and (**b**) LDO.

Figure 5 shows EDS analysis charts of LDHs and LDO. The percentage content of each element of LDHs and LDO is shown in Table 2. It can be seen from the figure that the samples mainly contain C, O, Mg, and Al elements, and there are more C and O contents in the samples before calcination, but the contents of C and O decrease while the contents of Mg and Al increase after calcination. This indicates that C and O escape in the form of CO_2_ and H_2_O, after roasting, and the product is MgO oxide at this time.

#### 4.1.4. The FTIR 4.1.4 The FT-IR Analysis of LDHs and LDO

**Figure 6:**
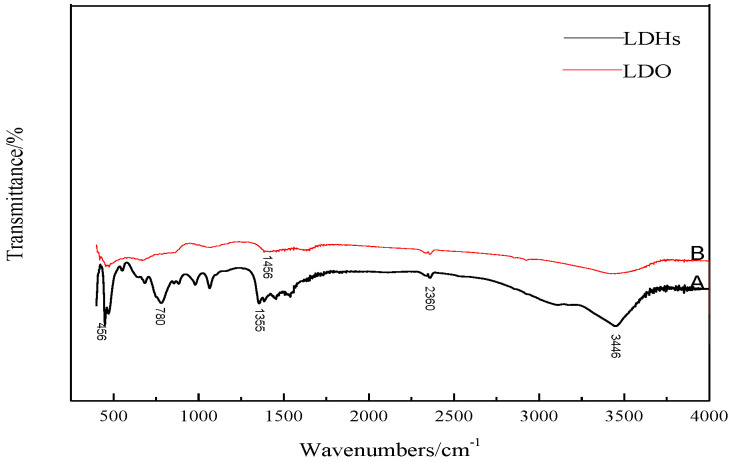
Infrared spectra of LDHs and LDO.

Figure 6 is an infrared spectrogram. It can be seen from the A curve that LDHs material has a strong and wide absorption peak at about 3446 cm^−1^, which is caused by the stretching vibration of hydroxyl group [3]. CO_3_^2−^ absorption peaks appear near 1355 and 780 cm^−1^, which indicates that the obtained material is a carbonate hydrotalcite–like material. The absorption peak at 456 cm^−1^ is the lattice vibration of cations (Mg^2+^, Al^3+^) [18]. B curve is the infrared spectrogram of LDO baked at 450 °C. Compared with the spectrogram of LDHs, the stretching vibration of hydroxyl group and the vibration absorption peak of CO_3_^2−^ are weakened, but they still exist, and CO_3_^2−^ is partially converted into CO_2_ gas, to form magnesium oxide.

### 4.2. Adsorption Results of LDO in Aqueous Solution

#### 4.2.1. Adsorption Results of LDO in Single Solution

##### Effect of Time on Adsorption Performance

Six 50 mL NaCl solutions with an initial concentration of 0.01 mol/L and six 50 mL Na_2_SO_4_ solutions with an initial concentration of 0.0075 mol/L were prepared, respectively, and the dosage of hydrotalcite was 0.2 g. The solutions were put into a constant temperature oscillator with a temperature of 35 °C and a rotating speed of 180 rpm, for contact reaction. The adsorbed solutions were taken and filtered every 1 h, and the concentrations of Cl^−^ and SO_4_^2−^ were measured. After sorting out the results, the adsorption capacity of LDO and the removal rate of Cl^−^ and SO_4_^2−^ were calculated.

**Figure 7:**
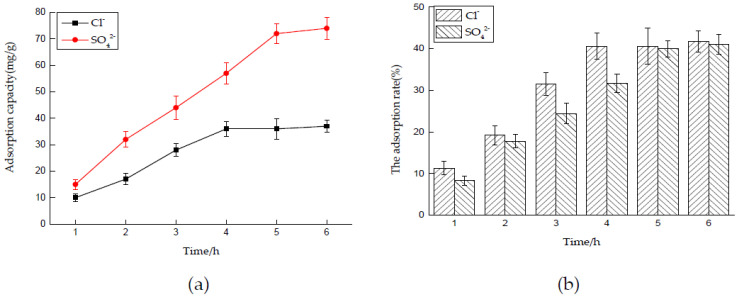
Effect of time on adsorption performance: (**a**) adsorption capacity and (**b**) adsorption rate.

The experimental results are shown in Figure 7a,b. It can be seen that the adsorption of Cl^−^ and SO_4_^2−^ by LDO is faster in the early stage, because there is a large number of active sites on the surface of LDO, in the early stage of reaction. When adsorption went on for 4 and 6 h, the concentration of Cl^−^ and SO_4_^2−^ in the solution basically did not change, and it can be considered that adsorption reached saturation state, at which time the adsorption site of LDO was completely occupied. Therefore, the best adsorption time for Cl^−^ and SO_4_^2−^ by LDO is 4 and 6 h, respectively.

##### Effect of Temperature on Adsorption Performance

Five 50 mL NaCl solutions with an initial concentration of 0.01 mol/L and five 50 mL Na_2_SO_4_ solutions with an initial concentration of 0.0075 mol/L were prepared, respectively, and the dosage of hydrotalcite was 0.2 g. The solutions were put into a constant temperature oscillator, at 25, 30, 35, 40, and 45 °C, respectively, and the rotating speed was 180 rpm for contact reaction. After 4 and 6 h, the adsorbed solutions were taken and filtered, and the concentrations of Cl^−^ and SO_4_^2−^ were measured. After sorting out the results, the adsorption capacity of LDO and the removal rate of Cl^−^ and SO_4_^2−^ were calculated.

**Figure 8:**
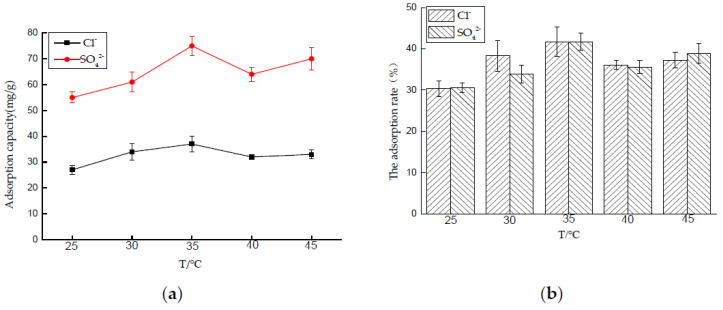
Effect of temperature on adsorption performance: (**a**) adsorption capacity and (**b**) adsorption rate.

In the adsorption process, ambient temperature is an important factor. As shown in Figure 8, from 25 to 35 °C, the adsorption capacity of LDO for Cl^−^ and SO_4_^2−^ increases rapidly with the increase of temperature, and it reaches the peak at 35 °C, and then decreases with the increase of temperature. The main reason is that, during the period from 25 to 35 °C, the temperature increase accelerates the migration of Cl^−^ and SO_4_^2−^ on the surface of LDO to its interior and releases the adsorption sites on the surface of LDO, which gradually enhances the adsorption capacity. Adsorption of Cl^−^ and SO_4_^2−^ by LDO can be divided into physical adsorption and chemical adsorption. With the further increase of temperature, chemical adsorption is dominant at this time. According to the principle of equilibrium shift, for exothermic reaction, the increase of temperature leads to the equilibrium shift to desorption direction; thus, the saturated adsorption capacity decreases gradually with the increase of temperature.

##### Effect of Dosage on Adsorption Performance

Five 50 mL NaCl solutions with an initial concentration of 0.01 mol/L and five 50 mL Na_2_SO_4_ solutions with an initial concentration of 0.0075 mol/L were prepared, respectively. The amounts of hydrotalcite are 0.225, 0.2, 0.175, 0.15, and 0.125 g respectively. The solutions were put into a constant temperature oscillator with a temperature of 35 °C and a rotating speed of 180 rpm, for contact reaction. After 4 and 6 h, the adsorbed solutions were taken and filtered, and the concentrations of Cl^−^ and SO_4_^2−^ were measured. After sorting out the results, the adsorption capacity of LDO and the removal rate of Cl^−^ and SO_4_^2−^ were calculated.

**Figure 9:**
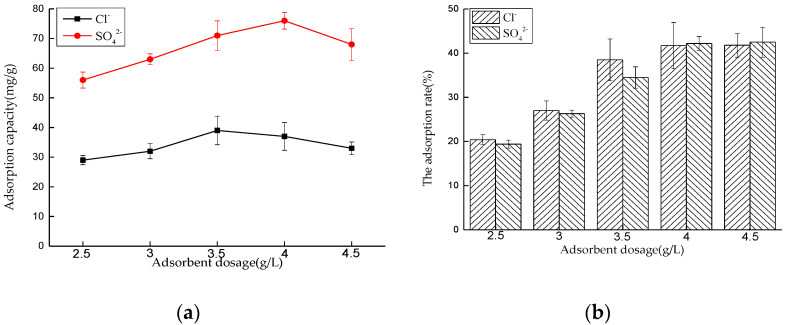
Effect of dosage on adsorption performance: (**a**) adsorption capacity and (**b**) adsorption rate.

The experimental results are shown in Figure 9. With the increase of LDO adsorption dose, the adsorption capacity of Cl^−^ and SO_4_^2−^ increases. When the solid–liquid ratio is 3.5 and 4.0 g/L, the adsorption capacity of Cl^−^ and SO_4_^2−^ by LDO is 39 and 76 mg/g, respectively. With the further increase of dosage, the adsorption capacity of Cl^−^ and SO_4_^2−^ by LDO decreases gradually. The performance of adsorbents is closely related to the specific surface area and active sites of the adsorbents. When the dosage is small, the contents of Cl^−^ and SO_4_^2−^ are sufficient, and LDO can reach saturation adsorption state. With the increase of dosage, the total amounts of Cl^−^ and SO_4_^2−^ in a certain volume of solution are limited, so Cl^−^ and SO_4_^2−^ cannot occupy more active sites of adsorbents, resulting in the decrease of adsorption capacity of Cl^−^ and SO_4_^2−^ per unit LDO adsorbent and the decrease of utilization rate of adsorbent. Therefore, it can be considered that the best LDO dosage for Cl^−^ adsorption is 3.5 g/L, and the best LDO dosage for SO_4_^2−^ adsorption is 4 g/L.

##### Effect of pH on Adsorption Performance

**Figure 10:**
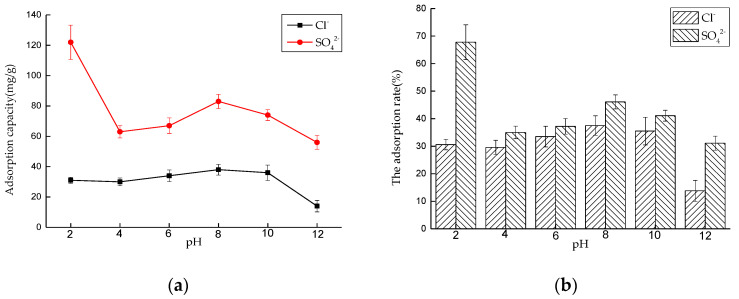
Effect of pH on adsorption performance: (**a**) adsorption capacity and (**b**) adsorption rate.

Six 50 mL NaCl solutions with an initial concentration of 0.01 mol/L and six 50 mL Na_2_SO_4_ solutions with an initial concentration of 0.0075 mol/L were prepared, respectively. We adjusted the pH value of the solutions to 2, 4, 6, 8, 10, and 12 with 0.1 mol/L NaOH and 0.1 mol/L HNO_3_, and added 0.175 and 0.2 g of LDO, respectively. The solutions were put into a constant temperature oscillator with a temperature of 35 °C and a rotating speed of 180 rpm, for contact reaction. After 4 and 6 h, the adsorbed solutions were taken and filtered, and the concentrations of Cl^−^ and SO_4_^2−^ were measured. After sorting out the results, the adsorption capacity of LDO and the removal rate of Cl^−^ and SO_4_^2−^ were calculated (Figure 10).

For Cl^−^, when the pH value is in the range of 2~10, the adsorption capacity of LDO for Cl^−^ does not change obviously with pH value, and it remains above 30mg/g, but when the pH value is greater than 10, its adsorption capacity decreases with the pH change. The main reason is that LDO has a memory effect in weak acid or weak alkali environment, and it will release OH^−^ when reconstructing the structure in water, which increases the alkalinity of the reaction system and plays a buffering role, so the adsorption amount of Cl^−^ by LDO does not change obviously with pH value under this condition. However, in a strong alkaline environment, LDO will undergo partial hydrolysis, which leads to a significant decline in its adsorption performance, which is unfavorable to the adsorption of Cl^−^.

For S0_4_^2−^, the pH value of the solution has a great influence on the adsorption performance. With the increase of acidity and alkalinity of the solution, the adsorption capacity of SO_4_^2−^ by LDO decreased, except for extremely acidic conditions (pH value is 2.0~4.0). When pH value drops from 4.0 to 2.0, adsorption capacity increases sharply. The adsorption capacity of SO_4_^2−^ is 122 mg/g when pH value is 2.0. Under the acidic condition with a pH value of 4.0~6.0, with the decrease of pH value, the amount of NO_3_^−^ increases, and the competitive action of NO_3_^−^ leads to the decrease of adsorption amount of SO_4_^2−^.Under the alkaline condition of pH 8.0~12.0, with the increase of alkalinity, the amount of OH^−^ in the solution increases, OH^−^ can compete with SO_4_^2−^ for adsorption sites, and the adsorption amount of SO_4_^2−^ decreases.

#### 4.2.2. Adsorption of LDO in Mixed Solution

##### Effect of Time on Adsorption Performance in Mixed Solution

Six 50 mL NaCl and Na_2_SO_4_ mixed solutions were prepared, and the concentrations of Cl^−^ and SO_4_^2−^ were similar. The amount of LDO is 0.4 g. The solutions were put into a constant temperature oscillator with a temperature of 35 °C and a rotating speed of 180 rpm, for contact reaction. The adsorbed solutions were taken and filtered every 1 h, and the concentrations of Cl^−^ and SO_4_^2−^ were measured. After sorting out the results, the adsorption capacity of LDO and the removal rate of Cl^−^ and SO_4_^2−^ were calculated.

**Figure 11:**
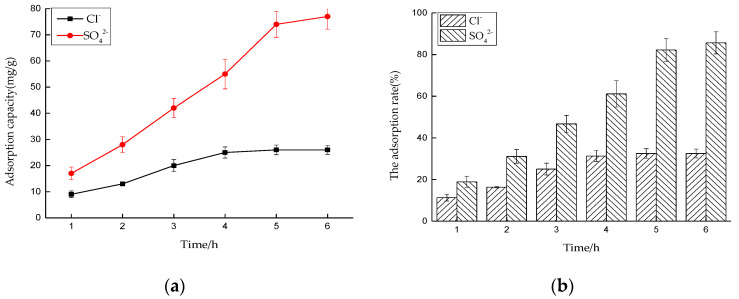
Effect of time on adsorption performance in mixed solution: (**a**) adsorption capacity and (**b**) adsorption rate.

It can be seen from the figure that the adsorption curves of Cl^−^ and SO_4_^2−^ by LDO in mixed solution are similar to those in Figure 7. Cl^−^ and SO_4_^2−^ reach saturated adsorption state in 4 and 6 h, respectively. However, the adsorption capacity of Cl^−^ in mixed solution is lower than that in single solution, and the maximum adsorption capacity is lower than 30 mg/g. The adsorption capacity of SO_4_^2−^ changes little and remains at a high level. Therefore, it can be preliminarily considered that the existence of other ions will have a great influence on the adsorption of Cl^−^, and SO_4_^2−^ is more easily adsorbed by LDO than Cl^−^ in mixed solution. In other experimental groups, in order to measure the maximum adsorption capacity of SO_4_^2−^, the adsorption time was set to 6 h (Figure 11).

##### Effect of Temperature on Adsorption Performance in Mixed Solution

Five 50 mL NaCl and Na_2_SO_4_ mixed solutions were prepared, and the concentrations of Cl^−^ and SO_4_^2−^ were similar. The amount of LDO is 0.4 g. The solutions were put into a constant temperature oscillator at 25, 30, 35, 40, and 45 °C, respectively, and the rotating speed was 180 rpm, for contact reaction. After 6 h, the adsorbed solutions were taken and filtered, and the concentrations of Cl^−^ and SO_4_^2−^ were measured. After sorting out the results, the adsorption capacity of LDO and the removal rate of Cl^−^ and SO_4_^2−^ were calculated.

**Figure 12:**
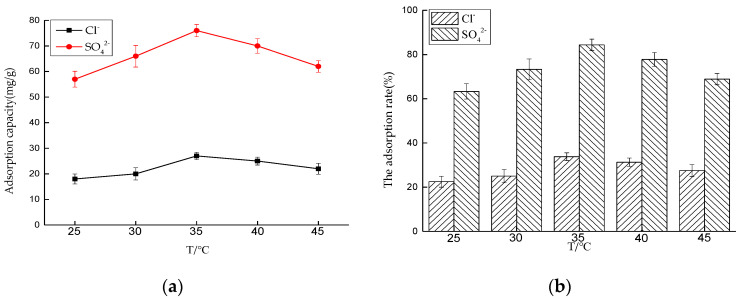
Effect of temperature on adsorption performance in mixed solution: (**a**) adsorption capacity and (**b**) adsorption rate.

As shown in Figure 12, the adsorption capacity of LDO for Cl^−^ reaches its peak at 35 °C, but it is lower than 30 mg/g. It proves that LDO preferentially adsorbs SO_4_^2−^ in the competitive adsorption of Cl^−^ and SO_4_^2−^ in mixed solution. For SO_4_^2−^, the solution temperature has a certain influence on its adsorption capacity. In the range of 25 to 35 °C, with the increase of temperature, its adsorption capacity increases steadily. When the temperature exceeds 35 °C, the saturated adsorption capacity decreases gradually with the increase of temperature.

##### Effect of Dosage on Adsorption Performance in Mixed Solution

Five 50 mL NaCl and Na_2_SO_4_ mixed solutions were prepared, and the concentrations of Cl^−^ and SO_4_^2−^ were similar. The amounts of hydrotalcite are 0.5, 0.45, 0.4, 0.35, and 0.3 g, respectively. The solutions were put into a constant temperature oscillator with a temperature of 35 °C and a rotating speed of 180 rpm, for contact reaction. After 6 h, the adsorbed solutions were taken and filtered, and the concentrations of Cl^−^ and SO_4_^2−^ were measured. After sorting out the results, the adsorption capacity of LDO and the removal rate of Cl^−^ and SO_4_^2−^ were calculated.

**Figure 13:**
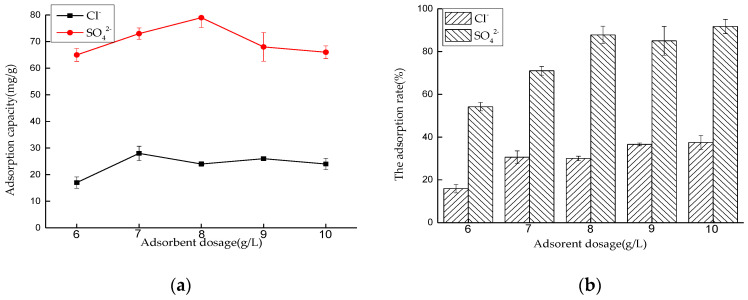
Effect of dosage on adsorption performance in mixed solution: (**a**) adsorption capacity and (**b**) adsorption rate.

It can be seen from Figure 13 that the adsorption capacity of LDO for SO_4_^2−^ is greater than that for Cl^−^ in the mixed solution. The optimal dosage of LDO to adsorb Cl^−^ and SO_4_^2−^ is 7 and 8 g/L, respectively. The experimental results show that higher valence anions are easier to combine with the active sites of LDO than monovalent anions. In other experimental groups, in order to measure the maximum adsorption capacity of SO_4_^2−^, the dosage of LDO was set to 8 g/L.

##### Effect of pH on Adsorption Performance in Mixed Solution

Six 50 mL NaCl and Na_2_SO_4_ mixed solutions were prepared, and the concentrations of Cl^−^ and SO_4_^2−^ were similar. We adjusted the pH value of the solution to 2, 4, 6, 8, 10, and 12 with 0.1 mol/L NaOH and 0.1 mol/L HNO_3_. The amount of LDO is 0.4 g. The solutions were put into a constant temperature oscillator with a temperature of 35 °C and a rotating speed of 180 rpm, for contact reaction. After 6 h, the adsorbed solutions were taken and filtered, and the concentrations of Cl^−^ and SO_4_^2−^ were measured. After sorting out the results, the adsorption capacity of LDO and the removal rate of Cl^−^ and SO_4_^2−^ were calculated.

**Figure 14:**
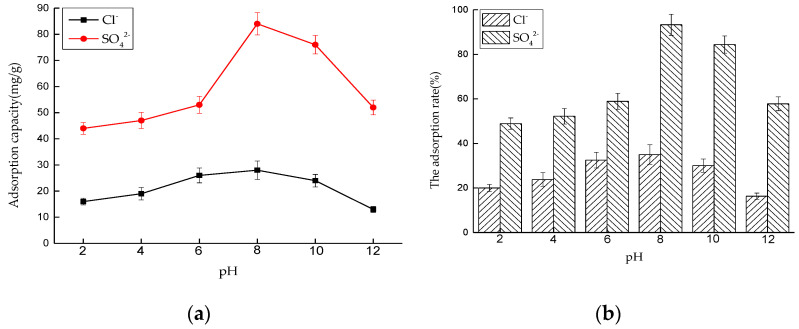
Effect of pH on adsorption performance in mixed solution: (**a**) adsorption capacity and (**b**) adsorption rate.

As shown in Figure 14, for Cl^−^, although the environment of peracid and peralkali has some influence on its adsorption capacity, the influence is not great. LDO preferentially adsorbs SO_4_^2−^, and the total adsorption capacity of Cl^−^ is still very small. Compared with Figure 10, the influence of pH on the adsorption capacity of SO_4_^2−^ in mixed solution is greater than that of SO_4_^2−^ in single solution. Especially, compared with the adsorption capacity in acidic environment, it can be found that the pH has a significant effect on the adsorption capacity of SO_4_^2−^ in the presence of Cl^−^.

### 4.3. Experiment of LDO in Cement

#### 4.3.1. Compressive Strength

**Figure 15:**
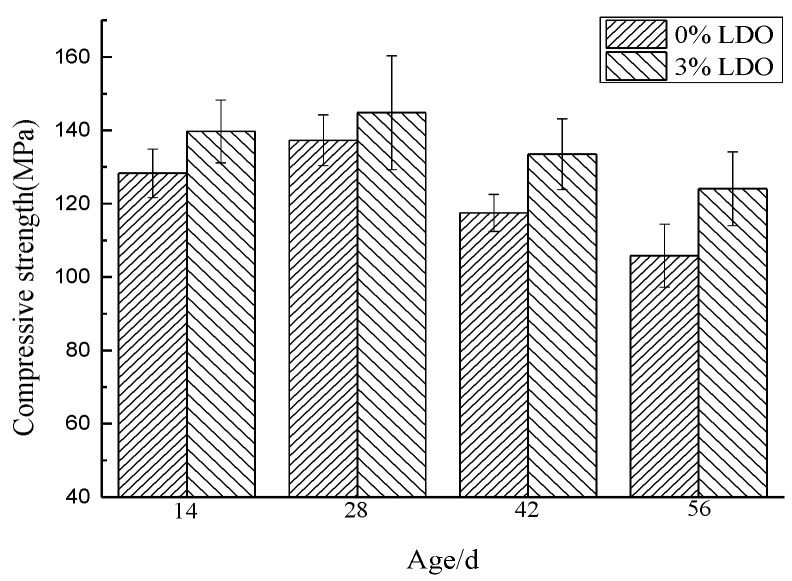
Compressive strength of the cement samples at different ages.

Figure 15 is a comparison chart of compressive strength of specimens at different ages after LDO is added into cement paste specimens. It can be seen from the figure that the incorporation of LDO has a certain influence on the strength when sulfate and chloride attack time is short, and the compressive strength of the experimental group and the control group at 14 days is 139.7 and 128.3 MPa, respectively. In the presence of sulfate, sulfate will react with the hydration products of cement, to produce ettringite, gypsum, and other expansive products, and the volume of the reaction products will fill some gaps in the specimen, so the strength of cement specimen will be improved to a certain extent. On the 28th day, the strength of the experimental group and the control group increased to 144.8 and 137.3 Mpa, respectively. However, if the reaction continues to occur, the continuous generation of intumescent products will lead to increasing expansion stress. When the expansion stress is greater than the tensile strength of the specimen, it will cause cracking of the specimen, which will lead to the decrease of its compressive strength. Therefore, with the aggravation of erosion, the strength of cement specimens will gradually decrease. At this time, however, the function of LDO gradually appeared. Compared with the control group, it can be seen that, although the intensity of the experimental group doped with LDO is decreasing, it is always higher than that of the control group without LDO. This shows that LDO adsorbs external ions, which reduces the formation of ettringite and slows down the strength decline trend of the specimen to some extent.

#### 4.3.2. Expansion Measurements

**Figure 16:**
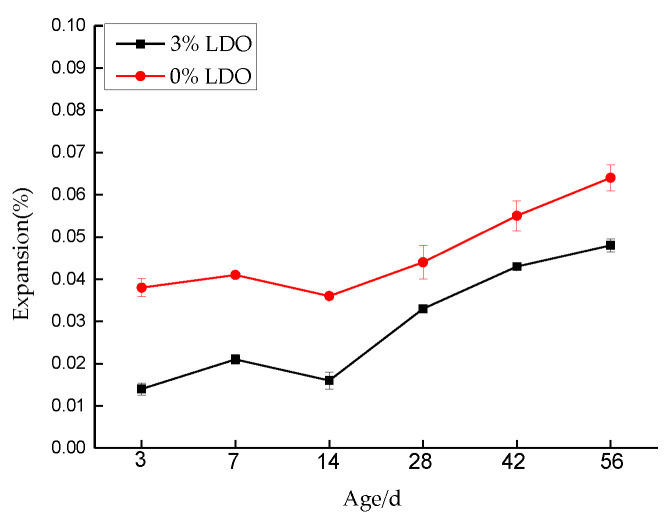
Expansion curves of cement samples.

Figure 16 is a comparison chart of expansion data of specimens at different ages, after LDO is added into cement paste specimens. It can be seen from the figure that no matter the experimental group with LDO or the control group without LDO, the expansion rate of the control group is only 0.064%, even after 56 days of erosion. However, it can be seen that the expansion of the experimental group is still smaller than that of the control group, which indicates that LDO in the cement paste specimen of the experimental group can well solidify external ions, inhibit the generation of expansive products, and thus maintain the volume stability of the specimen.

#### 4.3.3. TG–DSC Analysis

**Figure 17:**
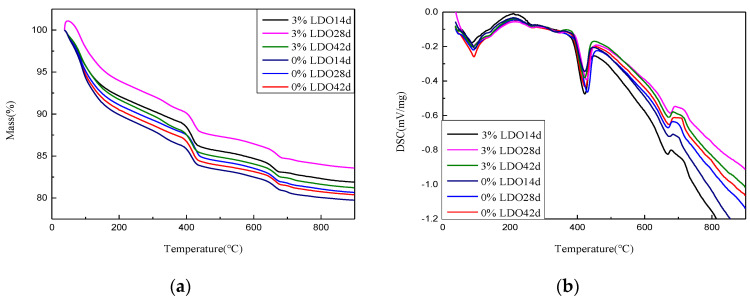
Thermogravimetry and differential scanning calorimetry (TG–DSC) curves of cement at different ages: (**a**) TG and (**b**) DSC.

In order to study the change of content of ettringite in cement paste specimens, samples at different ages were taken for TG–DSC analysis. The results are shown in Figure 17. The absorption peak at about 90 °C is due to the decomposition of ettringite, and the content of ettringite was calculated.

Table 3 shows the content of ettringite in each sample. It can be seen that the ettringite production of LDO-doped samples is significantly lower than that of the control group, which is consistent with the conclusion of macroscopic experiments.

## 5. Conclusions

The main conclusions are as follows:

1. LDHs is prepared by a simple hydrothermal synthesis method, and the structure and properties of the materials are analyzed by modern micro-analysis techniques, including phase analysis, morphology observation, and infrared spectroscopy. XRD analysis shows that the peak width of the product is narrow and sharp, there is no obvious impurity peak, and the product purity is high. From the electron microscope, it can be seen that the hexagonal LDHs prepared with ethanol as raw material have regular and uniform morphology; a cross-supporting structure is formed between the plates, and the dispersion is good. After roasting, the morphology of the samples has little change, and the original structure is still maintained. Infrared analysis shows that the unbaked LDHs material is carbonate hydrotalcite–like material. Compared with LDHs, the stretching vibration of hydroxyl group and the vibration absorption peak of CO_3_^2−^ are weakened, but they still exist, and CO_3_^2−^ is partially converted into CO_2_ gas to form magnesium–aluminum oxide. These microscopic test results confirm each other, which shows the reliability of the results and lays a foundation for the application of LDO materials in cement concrete.
2. The experimental results of LDO in separate solution show that the best conditions for Cl^−^ adsorption are an adsorption time of 4 h, temperature of 35 °C, LDO dosage of 3.5 g/L, and pH of 8. For SO_4_^2−^, the adsorption time is 6 h, temperature is 35 °C, LDO dosage is 4 g/L, and pH = 8; the adsorption effect is the best.
3. The experiment of LDO in mixed solution shows that LDO still has good adsorption effect in the system closer to the actual environment, but the adsorption of two kinds of ions shows some differences. Under the influence of various factors, the adsorption effect of LDO on SO_4_^2−^ is good, but it has a great influence on Cl^−^. The LDO preferentially adsorbs SO_4_^2−^ when both SO_4_^2−^ and Cl^−^ exist. When the amount of LDO is fixed and the ion concentration in the solution is high, a large amount of SO_4_^2−^ occupies the active sites of hydrotalcite, and the remaining active sites for Cl^−^ adsorption are less, which shows that the adsorption capacity of SO_4_^2−^ is larger than that of Cl^−^. However, the adsorption capacity of LDO for these two ions in the actual system is less than that in single ion solution. This may be due to the existence of a small amount of other ions in the mixed system, which will compete with SO_4_^2−^ and Cl^−^ for adsorption, thus reducing the adsorption capacity of SO_4_^2−^ and Cl^−^.
4. The experiment of LDO in cement shows that, compared with ordinary cement paste specimens, the specimens doped with LDO have less of an ettringite formation, a stronger ability to cure SO_4_^2−^ and Cl^−^, and the LDO enhances the compressive strength and stability of the specimens.

## Figures and Tables

**Table 1:** Chemical compositions of P·II 52.5 Portland cement.

SiO_2_	CaO	Al_2_O_3_	Fe_2_O_3_	MgO	Na_2_O	SO_3_
19.58	63.85	4.63	2.91	2.45	0.14	3.27

**Table 2:** The percentage content of each element of LDHs and LDO.

Samples	Element	Weight/%	Atom/%
LDHs	C	16.92	24.20
	O	49.40	53.05
	Mg	18.68	13.20
	Al	15.00	9.55
LDO	C	4.04	6.75
	O	37.39	47.52
	Mg	31.89	25.90
	Al	26.68	19.83

**Table 3:** The content of ettringite at different ages.

Sample	0%LDO 14 d	0%LDO 28 d	0%LDO 42 d	3%LDO 14 d	3%LDO 28 d	3%LDO 42 d
Ettringite (%)	11.14	12.53	14.24	3.57	8.51	9.1

## Data Availability

Data sharing is not applicable.

## References

[1] Zhang L. (2018). Synthesis of Mg–Al Layered Double Hydroxides and Their Adsorption Capability of Chloride Ion in Cement Paste. Ph.D. Thesis.

[2] Gong X. (2008). The Experimental Research of Sulfate Attack Resistance of Concrete. Ph.D. Thesis.

[3] Duan P. (2014). Research on Modification Mechanism and the Application of Layered Double Hydroxides for Durability of Concrete. Ph.D. Thesis.

[4] Mo D. (2004). Preferential Intercalation in Layered Double Hydroxides and Mechanism Study. Ph.D. Thesis.

[5] Evans D.G., Slade R., Duan X., Evans D.G. (2006). Structural Aspects of layered double hydoxides. Structure and Bonding.

[6] Li F., Duan X. (2006). Applications of layered double hydroxides. Structure and Bonding.

[7] Jone L. (1988). Process for Removing Heavy Metal Ions from Solutions Using Adsorbents Containing Activated Hydrotalcite.

[8] Orthman J., Zhu H., Lu G. (2003). Use of anion clay hydrotalcite to remove coloured organics from aqueous solutions. Sep. Purif. Technol..

[9] Ulibarri M., Pavlovic I., Hermosín M., Cornejo J. (1995). Hydrotalcite-like compounds as potential sorbents of phenols from water. Appl. Clay Sci..

[10] Tatematsu H., Akamura T., Koshimuzu H., Morishita T., Kotaki H. (1996). Cement additive for inhibiting concrete deteriora-tion. Ziolites.

[11] Shui Z., Ma J., Chen W., Chen X. (2012). Chloride Binding Capacity of Cement Paste Containing Layered Double Hydroxide (LDH). J. Test. Eval..

[12] Shui Z., Ma J. (2012). The effect of layered double hydroxides on the concrete resistance of chloride-ion penetration. Key Eng. Mater..

[13] Ke X., Bernal S.A., Provis J.L. (2017). Uptake of chloride and carbonate by Mg-Al and Ca-Al layered double hydroxides in simulated pore solutions of alkali-activated slag cement. Cem. Concr. Res..

[14] Yang L., Chen M., Lu Z., Huang Y., Wang J., Lu L., Cheng X. (2020). Synthesis of CaFeAl layered double hydroxides 2D nanosheets and the adsorption behaviour of chloride in simulated marine concrete. Cem. Concr. Compos..

[15] Yoon S., Moon J., Bae S., Duan X., Giannelis E.P., Monteiro P.M. (2014). Chloride adsorption by calcined layered double hydroxides in hardened Portland cement paste. Mater. Chem. Phys..

[16] Chen Y., Shui Z., Chen W., Chen G. (2015). Chloride binding of synthetic Ca–Al–NO3 LDHs in hardened cement paste. Constr. Build. Mater..

[17] Yang Z., Fischer H., Polder R. (2015). Laboratory investigation of the influence of two types of modified hydrotalcites on chloride ingress into cement mortar. Cem. Concr. Compos..

[18] Tong M. (2014). Preparation and performance study on Mg–Al-hydrotalcite. Inorg. Chem. Ind..

